# Influence of Stochastic Resonance on Manual Dexterity in Children With Developmental Coordination Disorder: A Double-Blind Interventional Study

**DOI:** 10.3389/fneur.2021.626608

**Published:** 2021-03-30

**Authors:** Satoshi Nobusako, Michihiro Osumi, Atsushi Matsuo, Emi Furukawa, Takaki Maeda, Sotaro Shimada, Akio Nakai, Shu Morioka

**Affiliations:** ^1^Neurorehabilitation Research Center, Kio University , Kitakatsuragi-gun, Japan; ^2^Graduate School of Health Science, Kio University, Kitakatsuragi-gun, Japan; ^3^Faculty of Nursing, University of Hyogo, Akashi, Japan; ^4^Department of Neuropsychiatry, Keio University School of Medicine, Tokyo, Japan; ^5^Department of Electronics and Bioinformatics School of Science and Technology, Meiji University, Kawasaki, Japan; ^6^Graduate School of Clinical Education & The Center for the Study of Child Development, Institute for Education, Mukogawa Women's University, Nishinomiya, Japan

**Keywords:** children, developmental coordination disorder, double-blind study, manual dexterity, stochastic resonance, subthreshold vibrotactile noise stimulation

## Abstract

**Background:** There is increasing evidence that the stochastic resonance (SR) phenomenon provided by subthreshold mechanical noise stimulation improves the sensory-motor system. However, the effect of SR on children with developmental coordination disorder (DCD) is unclear. The purpose of this study was to assess whether SR activated by subthreshold vibrotactile noise stimulation of the wrist influences manual dexterity in children with DCD.

**Methods:** A double-blind interventional study was conducted. Participants were 30 children (age: 9.3 ± 1.44 years, range 6–11 years; 27 male, three female; 25 right-handed, five left-handed) meeting DCD diagnostic criteria in DSM-5. The manual dexterity test was administered the day before SR intervention (baseline-data). SR was elicited using subthreshold vibrotactile noise stimulation at 60% of the vibrotactile threshold measured at the wrist. SR was delivered two times and the manual dexterity test was administered during each SR stimulation block (SR-on condition) and after each SR stimulation block (SR-off), for a total of four measurements. Target outcomes were the component score, the standard score, and the percentile score of the manual dexterity test.

**Results:** The manual dexterity test scores in the SR-on condition were significantly improved compared to scores at the baseline and in the SR-off condition (*p* < 0.001).

**Conclusions:** The present study showed that subthreshold noise stimulation eliciting SR significantly improved manual dexterity outcomes in children with DCD during stimulation but not after stimulation. Future studies will need to investigate the carry-over effects of SR stimulation.

## Introduction

Developmental coordination disorder (DCD) is characterized by clumsiness in fine (hand writing and shoelace tying) and gross (playing sport and getting dressed) motor skills and affects ~5–6% of school-aged children, making it the most common childhood movement disorder (1, 2). DCD in children not only affects daily life performance, but also has psychological implications such as reduced self-esteem and increased risk of anxiety and depression (3–5). In 50–70% of children with DCD, motor difficulties persist through adolescence and adulthood (1, 2). Therefore, motor difficulties are an important concern and the development of effective intervention is an urgent issue.

Subthreshold mechanical noise stimulation to the body is known to improve the sensory-motor system. This improvement is related to the stochastic resonance (SR) phenomenon, also known as “noise benefit,” that can occur in various sensory and motor systems (6). For example, SR has been shown to improve tactile sensitivity (7, 8). In addition, previous studies have demonstrated improvements in balance, walking, and hand movements due to SR elicited by vibrotactile noise stimulation (9–11). These kinds of improvements were observed not only in healthy participants but also in patients with diabetes, stroke, and Parkinson's disease, and in children with cerebral palsy (7–10, 12, 13).

One case study reported the effect of SR on a child with DCD (14). This study showed that the manual dexterity test score during SR was significantly improved compared to the score obtained without SR, suggesting that stimulation eliciting SR could be effective for children with DCD. However, the study was a case study and further investigation is needed to address the possible effect of SR on clumsiness in children with DCD. In this study we addressed the influence of SR on manual dexterity in children with DCD. We used a block design with / without SR in a double-blind intervention study, with both children and evaluators blinded to the SR condition.

## Materials and Methods

### Participants

Children were enrolled in regular classes at public primary schools in Osaka, Japan, and were recruited from the pool of children who were requested physical assessment and physical therapy due to clumsiness by their teachers or parents. Participants met the four DCD diagnostic criteria (A–D) in the Diagnostic and statistical manual of mental disorders 5th edition (DSM-5) (1): (A) Less than the 16th percentile in the Movement Assessment Battery for Children-2nd Edition (M-ABC2); (B) Less than the cut-off point of the Japanese version of the Developmental Coordination Disorder Questionnaire (DCDQ) (15), (C) Onset of symptoms early in development, and (D) No diagnosis of a general medical condition (e.g., cerebral palsy, hemiplegia, and muscular dystrophy), visual impairment, or intellectual disability (1). Eligibility was assessed by combining interviews to parents and the results of regular assessments provided by the school's doctor. Based on these four criteria, 30 children with DCD were selected (mean age ± standard deviation (SD): 9.3 ± 1.44 years; age range: 6–11 years; 27 male, three female; 25 right-handed, five left-handed). Although it was not an exclusion criterion, none of the children who participated in this study had a diagnosis of other developmental disorders (e.g., autism spectrum disorder, attention deficit hyperactivity disorder, and learning disorder). Table 1 shows the information of participated children collected the day before the SR intervention in this study (Table 1).

**Table 1:** Results of tests conducted on the day before the current study (Baseline-data).

**No**.	**Group**	**Age (years)**	**Sex**	**Preferred hand**	**M-ABC2**												**DCDQ**				**SCQ**	**ADHD-RS (%)**	**DSRS-C**
					**MD Component score**	**MD Standard score**	**MD Percentile score**	**A&C Component score**	**A&C Standard score**	**A&C Percentile score**	**Balance Component score**	**Balance Standard score**	**Balance Percentile score**	**Total Component score**	**Total Standard score**	**Total Percentile score**	**Control during movement**	**Fine motor and handwriting**	**General coordination**	**Total score**			
1	A	11	M	R	12	3	1	7	2	0.5	12	3	1	31	2	0.5	8	4	5	17	15	89	5
2	B	10	M	R	21	6	9	13	6	9	28	9	37	62	6	9	24	8	21	53	6	50	7
3	A	7	M	L	15	4	2	8	2	0.5	13	4	2	36	2	0.5	8	6	5	19	19	95	9
4	B	10	M	R	17	5	5	17	9	37	22	6	9	56	5	5	11	7	9	27	26	93	10
5	A	10	M	R	16	5	5	17	9	37	22	6	9	56	5	5	10	9	10	29	20	91	13
6	B	8	M	R	19	6	9	14	7	16	29	9	37	62	6	9	20	7	19	46	4	80	8
7	A	9	M	R	16	5	5	16	8	25	28	9	37	60	6	9	18	8	20	46	5	75	10
8	B	10	M	R	32	11	63	12	5	5	16	5	5	60	6	9	14	8	7	29	9	87	3
9	A	8	M	R	18	5	5	10	4	2	16	5	5	44	4	2	11	7	11	29	10	25	8
10	B	11	M	R	19	6	9	10	4	2	11	3	1	40	3	1	17	12	12	41	14	80	3
11	A	11	M	R	19	6	9	10	4	2	11	3	1	40	3	1	18	8	15	41	9	50	6
12	B	7	M	R	24	8	25	8	2	0.5	25	8	25	57	6	9	9	16	13	38	5	50	9
13	A	10	M	R	26	9	37	10	4	2	25	8	25	61	6	9	12	11	7	30	17	97	16
14	B	8	M	L	21	6	9	11	5	5	30	9	37	62	6	9	15	8	8	31	25	99	13
15	A	9	M	L	19	6	9	13	4	2	29	9	37	61	6	9	18	9	9	36	22	97	15
16	B	10	M	R	27	9	37	14	7	16	20	6	9	61	6	9	16	11	14	41	9	50	3
17	A	8	F	R	14	4	2	7	2	0.5	10	2	0.5	31	2	0.5	11	11	11	33	1	50	3
18	B	9	M	R	21	6	9	11	5	5	15	5	5	47	4	2	15	7	16	38	4	89	5
19	A	8	M	R	21	6	9	11	5	5	15	5	5	47	4	2	12	8	15	35	6	91	7
20	B	11	M	R	21	6	9	12	5	5	25	8	25	58	6	9	13	16	13	42	9	50	12
21	A	11	F	R	6	2	0.5	9	3	1	9	2	0.5	24	1	0.1	10	12	11	33	4	94	7
22	B	6	M	L	10	3	1	19	10	50	19	6	9	48	4	2	13	6	7	26	2	95	1
23	A	7	M	L	10	3	1	19	10	50	19	6	9	48	4	2	12	4	7	23	10	98	4
24	B	10	M	R	20	6	9	15	8	25	27	8	25	62	6	9	18	12	15	45	2	25	8
25	A	9	M	R	20	6	9	18	9	37	22	6	9	60	6	9	20	17	14	51	1	50	8
26	B	8	M	R	19	6	9	8	2	0.5	28	9	37	55	5	5	12	4	8	24	16	80	4
27	A	11	M	R	20	6	9	11	3	1	10	2	0.5	41	3	1	13	14	10	37	7	98	22
28	B	10	F	R	7	2	0.5	10	4	2	9	2	0.5	26	1	0.1	8	7	11	26	16	92	10
29	A	10	M	R	14	4	2	12	5	5	23	7	16	49	4	2	18	8	9	35	1	80	7
30	B	11	M	R	19	6	9	14	7	16	29	9	37	62	6	9	21	9	11	41	4	84	6
Mean		9.3	M, *n* = 27 F, *n* = 3	R, *n* = 25 L, *n* = 5	18.1	5.5	10.6	12.2	5.3	12.2	19.9	6.0	15.2	50.2	4.5	5.0	14.2	9.1	11.4	34.7	9.9	76.1	8.1
SD		1.4			5.5	2.0	13.2	3.4	2.5	15.2	7.0	2.4	14.1	11.7	1.6	3.7	4.2	3.4	4.1	8.9	7.3	22.3	4.5
Range		6–11			6–32	2–11	1–63	7–19	2–10	1–50	9–30	2–9	1–37	24–62	1–6	0.1–9	8–24	4–17	5–21	17–53	1–26	25–99	1–22
Skewness		−0.47			−0.07	0.51	2.57	0.45	0.39	1.32	−0.14	−0.25	0.55	−0.76	−0.68	0.02	0.39	0.69	0.57	0.04	0.67	−0.90	1.03
Kurtosis		−0.78			0.74	1.14	7.99	−0.64	−0.92	0.72	−1.44	−1.22	−1.35	−0.51	−0.72	−1.92	−0.62	0.09	−0.09	−0.49	−0.53	−0.35	1.75

*No., Reception number. The odd number of reception numbers was divided into Group A, and the even number was divided into Group B. M, Male; F, Female; R, Right; L, Left; M-ABC2, the Movement Assessment Battery for Children-2nd Edition; MD, Manual Dexterity; A&C, Aiming & Catching; DCDQ, the Developmental Coordination Disorder Questionnaire; SCQ, the Social Communication Questionnaire; ADHD-RS, the Attention-Deficit Hyperactivity Disorder-Rating Scale; DSRS-C, the Depression Self-Rating Scale for Children; SD, Standard Deviation*.

The experimental procedures were approved by the ethics committee of the Graduate School and Faculty of Health Sciences at Kio University (approval number: R1-22). There were no potential risks for study participants. No personal identification information was collected. Children and their parents and caregivers were given detailed explanation of the study and parents/caregivers provided written informed consent for participation of their children in the study and for publication of the study results. The experimental procedures were compliant with the ethical standards of the 1964 Declaration of Helsinki regarding the treatment of human participants in research.

### Procedures

**Figure 1:**
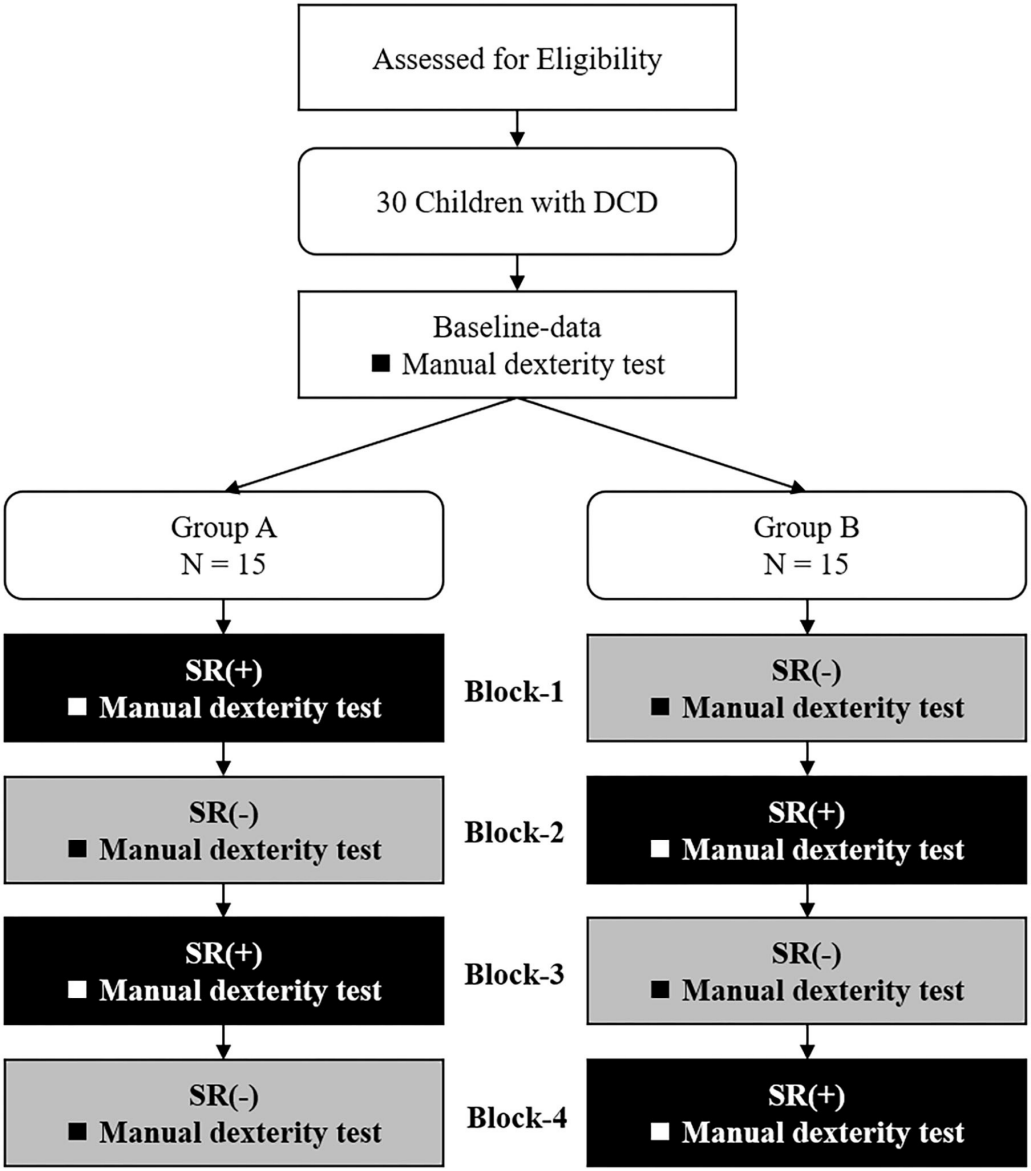
Current experimental protocol. DCD, developmental coordination disorder; SR(+), stochastic resonance-on condition; SR(-), stochastic resonance-off condition.

Figure 1 shows the experimental protocol. The study was a double-blind intervention study with block design. Experiments were conducted in the prescribed rooms at each primary school and were organized in two separate sessions in two subsequent days per each participant. On the first day, the M-ABC2 and other measurements (DCDQ, the Social Communication Questionnaire: SCQ, the Attention-Deficit Hyperactivity Disorder-Rating Scale: ADHD-RS, and the Depression Self-Rating Scale for Children: DSRS-C) were taken as the baseline-data. On the 2nd day, SR was delivered two times and the manual dexterity tests were administered during each SR stimulation block (SR-on condition) and after each SR stimulation block (SR-off), for a total of four measurements. To mitigate possible learning effects in the manual dexterity test, the participants were divided into two groups (A and B) according to their order of enrollment. Fifteen children (those with odd progressive number) were administered the manual dexterity test in the following order: SR-on, SR-off, SR-on, and SR-off conditions (Group A; age: 9.3 ± 1.39 years; range: 7–11 years; 13 male, two female; 12 right-handed, three left-handed). The remaining 15 children (those with even progressive numbers) were administered the manual dexterity test in the following order: SR-off, SR-on, SR-off, and SR-on conditions (Group B; age: 9.3 ± 1.48 years; range: 6–11 years; 14 male, one female; 13 right-handed, two left-handed). Participants and evaluators performing the manual dexterity test were blinded about the group participants were assigned to and were not aware of the SR-on and SR-off conditions. There were no significant differences in age (Z = −0.064, *p* = 0.967), sex [χ2 = 0.370, χ2_(0.95)_ = 3.841, *p* = 0.543], or preferred hand [χ2 = 0.240, χ2_(0.95)_ = 3.841, *p* = 0.624] between Group A and Group B.

### Stochastic Resonance Intervention: Subthreshold Vibrotactile Noise Stimulation

To elicit SR, subthreshold vibrotactile noise stimulation was applied using four compact devices (length: 10 mm; width: 18 mm; height: 2 mm; Vibration Actuator Sprinter α; Nidec Seimitsu, Nagano, Japan) attached to the volar and dorsal areas of children's right and left wrists using contact tape (i.e., two devices on the right wrist and two devices on the left wrist). The resonance frequency of the device was 170 ± 10 Hz (average ± SD). Low-pass filters at 500 Hz were used as per previous studies (7, 8, 10, 11, 14, 16, 17). A digital amplifier (FX Audio D802; North Flat Japan, Osaka, Japan) was used to output the white noise signals needed to elicit SR through the vibrotactile actuators. Consistent with previous studies (7, 8, 10, 11, 14, 16, 17), we attached the device to the wrist to minimize manual interruption while affecting the tactile sensation of the fingers. The intensity of the vibrotactile noise was set to 60% of the vibrotactile threshold at the beginning of the test—a level shown to be optimum to elicit SR in sensory systems (7, 8, 10, 11, 14, 16, 17). The sensory thresholds for vibrotactile noise were measured immediately before each of the four measurement blocks, irrespective of whether it was an SR-on or SR-off condition. The vibrotactile noise device remained attached throughout testing and it was turned on and off at the beginning and at the end of each block to implement the SR-on/SR-off conditions. Control over the SR-on/SR-off condition was given to an experimenter different than the one who administered the manual dexterity test. Children were blinded to the condition as they could not feel the subthreshold noise stimuli and were not informed about the SR-on/SR-off conditions.

### Outcome: Manual Dexterity Test

The manual dexterity test of the M-ABC2 is a standardized, age-adjusted test to evaluate the DCD diagnostic criterion A of DSM-5 (18). This test has good test-retest reliability (minimum value at any age is 0.75), good inter-rater reliability (0.70), and good concurrent validity (18). This test has three age bands: 3–6 years (age band 1), 7–10 years (age band 2), and 11–16 years (age band 3). In the current study, each child received three sub-tests that were appropriate for his/her age band. Children in the age band 1 (3–6 years) were evaluated by the posting coins test, threading beads test, and drawing trail I test. The age band 1 test was performed by 0 participants in Group A and 1 participant in Group B. Children in the age band 2 (7–10 years) were evaluated by the placing pegs test, threading lace test, and drawing trail II test. The age band 2 test was administered to 11 participants in Group A and 11 participants in Group B. Children in the age band 3 (11–16 years) were evaluated by the turning pegs test, triangle with nuts and bolts test, and drawing trail III test. The age band 3 test was completed by four participants in Group A and three participants in Group B. The manual dexterity test was conducted twice in each block, with a total of 8 tests for each child throughout the experiment. The component score, standard score, and percentile score were then calculated from the obtained raw scores. An increase in the component score, standard score, and percentile score suggest an improvement in manual dexterity. The manual dexterity test was administered by a specifically trained, certified physical therapist who was blinded to the SR-on and SR-off conditions.

### Statistical Analysis

The baseline data (age, sex, preferred hand, and M-ABC2, DCDQ, SCQ, ADHD-RS, and DSRS-C scores) were compared statistically between Groups A and B. Sex and preferred hand were compared between Groups A and B using the chi-square test for independence. According to the Shapiro-Wilk test, age, all percentile scores of M-ABC2, the control during movement of DCDQ, SCQ, and ADHD-RS scores were not normally distributed, so they were compared between Groups A and B using the Mann-Whitney U test. The DCDQ fine motor and handwriting, general coordination, and total scores, as well as the DSRS-C score, were normally distributed according to the Shapiro-Wilk test, and were compared between Groups A and B using an independent samples *t*-test.

The results of the manual dexterity test scores (component score, standard score, and percentile score) measured throughout the experiment (baseline-data, Block-1,−2,−3,−4) were compared in each of the two groups (Group A, *N* = 15; Group B, *N* = 15). The Shapiro-Wilk test showed that Group A component scores and standard scores and Group B component scores were normally distributed. Repeated measures one-way analysis of variance (ANOVA) was used to analyze these scores measured throughout the experiment (baseline-data, Block-1,−2,−3,−4). Multiple comparisons in *post-hoc* analyses were performed using paired *t*-tests. Group A percentile scores and Group B standard scores and percentile scores measured throughout the experiment (baseline-data, Block-1,−2,−3,−4) were compared using the Friedman test because those data were not normally distributed as shown by the Shapiro-Wilk test, and the Wilcoxon signed-rank test was used for multiple comparisons in *post-hoc* analyses.

The results of the manual dexterity test (component score, standard score, and percentile score) were compared considering the baseline-data, SR-on condition (scores averaged over the two blocks), and SR-off condition (scores averaged over the two blocks) in the whole group (*N* = 30). Since component scores were normally distributed as shown by the Shapiro-Wilk test, they were compared using repeated measures one-way ANOVA and *post-hoc* multiple comparisons were performed using paired *t*-tests. The standard scores and percentile scores were not normally distributed as shown by the Shapiro-Wilk test, they were compared using the Friedman test and *post-hoc* multiple comparisons were performed using the Wilcoxon signed-rank test.

The significance level was set at α = 0.05 for all statistical analyses, and the Bonferroni correction was used to adjust for multiple comparisons in *post-hoc* analyses. The effect size was calculated. All statistical analyses were performed using SPSS, version 24 (SPSS, Chicago, IL, USA).

## Results

Table 2 shows the results of the comparisons of baseline data between Groups A and B. There were no differences in age (*Z* = −0.064, *p* = 0.967), sex [χ^2^ = 0.370, $\chi_{\left(0.95\right)}^{2}$ = 3.841, *p* = 0.543], and preferred hand [χ^2^ = 0.240, $\chi_{\left(0.95\right)}^{2}$ = 3.841, *p* = 0.624] between Groups A and B. There were no differences in the M-ABC2 scores between Groups A and B (Manual dexterity: *Z* = −1.952, *p* = 0.067; Aiming and catching: *Z* = −1.154, *p* = 0.267; Balance: *Z* = −1.809, *p* = 0.074; Total: *Z* = −2.045, *p* = 0.050), but they tended to be slightly higher in Group B. There were no differences in the scores for DCDQ (Control during movement: *Z* = −1.188, *p* = 0.250; Fine motor and handwriting: *t* = −0.104, *p* = 0.918; General coordination: *t* = −1.095, *p* = 0.283; Total: *t* = −1.096, *p* = 0.283), SCQ (*Z* = −0.062, *p* = 0.967), ADHD-RS (*Z* = −0.983, *p* = 0.345), and DSRS-C (*t* = 1.563, *p* = 0.129) between Groups A and B.

**Table 2:** Comparisons of baseline data between Groups A and B.

**Group**		**Age (years)**	**Sex**	**Preferred hand**	**M-ABC2**				**DCDQ**				**SCQ**	**ADHD-RS**	**DSRS-C**
					**Manual dexterity**	**Aiming and catching**	**Balance**	**Total**	**Control during movement**	**Fine motor and handwriting**	**General coordination**	**Total**			
A	Mean	9.3	M, *n* = 13 F, *n* = 2	R, *n* = 12 L, *n* = 3	7.0	11.4	10.5	3.5	13.3	9.1	10.6	32.9	9.8	78.7	9.3
	SD	1.39			8.65	16.32	12.29	3.50	3.89	3.40	3.95	8.88	6.97	22.65	4.96
B	Mean	9.3	M, *n* = 14 F, *n* = 1	R, *n* = 13 L, *n* = 2	14.2	12.9	19.9	6.4	15.1	9.2	12.3	36.5	10.1	73.6	6.8
	SD	1.48			15.72	14.01	14.32	3.39	4.28	3.39	4.11	8.50	7.52	21.69	3.49
*P*-value		0.967	0.543	0.624	0.067	0.267	0.074	0.050	0.250	0.918	0.283	0.283	0.967	0.345	0.129

*The M-ABC2 and ADHD-RS scores are percentile score*.

*ADHD-RS, Attention-Deficit Hyperactivity Disorder-Rating Scale; DCDQ, Developmental Coordination Disorder Questionnaire; DSRS-C, Depression Self-Rating Scale for Children; F, Female; L, Left; M, Male; M-ABC2, Movement Assessment Battery for Children-2nd Edition; R, Right; SCQ, Social Communication Questionnaire; SD, Standard Deviation*.

All participants (*N* = 30) completed the current experimental protocol.

Statistical analysis showed that the changes in component scores observed in Group A and B throughout the experiment were statistically significant [Group A: *F*_(4, 56)_ = 8.156, *p* < 0.001; Group B: *F*_(4, 56)_ = 8.575, *p* < 0.001]. *Post-hoc* analysis showed that the component scores of Group A in Block-1 (SR-on condition) and Block-3 (SR-on condition) were significantly higher than in the baseline-data and Block-2 (SR-off condition) (Block-1 vs. baseline-data, *p* = 0.023; Block-1 vs. Block-2, *p* = 0.003; Block-3 vs. baseline-data, *p* = 0.038; Block-3 vs. Block-2, *p* = 0.027; all with after Bonferroni correction). In Group B, the component scores in Block-2 (SR-on condition) were significantly higher than the baseline-data (*p* = 0.008; after Bonferroni correction), and the component scores measured in Block-4 (SR-on condition) were significantly higher than the those measured at the baseline-data and in Block-1 (SR-off condition)(Block-4 vs. baseline-data, *p* = 0.001; Block-4 vs. Block-1, *p* = 0.036; after Bonferroni correction).

**Figure 2:**
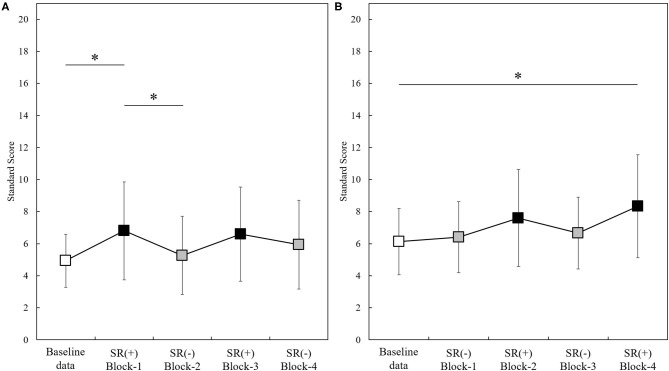
Standard scores measured through the experiment in the two groups (Group A, *N* = 15; Group B, *N* = 15). Baseline-data (white squares); SR(+): SR-on condition (black squares); SR(-): SR-off condition (gray squares). *p < 0.05. Panel **(A)**: Standard scores (mean ± standard deviation) in Group A (*N* =15). Panel **(B)**: Standard scores (mean ± standard deviation) in Group B (*N* =15).

Figure 2 shows the change standard scores for Group A (*N* = 15) and Group B (*N* = 15) throughout the experiment (baseline-data,−1,−2,−3,−4). The change in standard scores in Group A and B observed throughout the experiment (baseline-data, Block-1,−2,−3,−4) were statistically significant [Group A: *F*_(4, 56)_ = 7.204, *p* < 0.001; Group B: *p* < 0.001]. *Post-hoc* analysis showed that the standard scores measured in Group A in Block-1 (SR-on condition) were significantly higher than those at the baseline-data and those in Block-2 (SR-off condition) (Block-1 vs. baseline-data, *p* = 0.040; Block-1 vs. Block-2, *p* = 0.015; all Bonferroni-corrected) (Figure 2A). Standard scores measured in Group B in Block-4 (SR-on condition) were significantly higher than baseline-data (*p* = 0.020; after Bonferroni correction) (Figure 2B).

The change in percentile scores in Group A and B observed throughout the experiment (baseline-data, Block-1,−2,−3,−4) was significant (Group A: *p* < 0.001; Group B: *p* < 0.001). *Post-hoc* analysis showed that percentile scores measured in Group A in Block-1 (SR-on condition) were significantly higher than in Block-2 (SR-off condition) (*p* = 0.048; after Bonferroni correction), and that percentile scores measured in Group B in Block-4 (SR-on condition) were significantly higher than those from baseline-data (*p* = 0.022; after Bonferroni correction).

**Figure 3:**
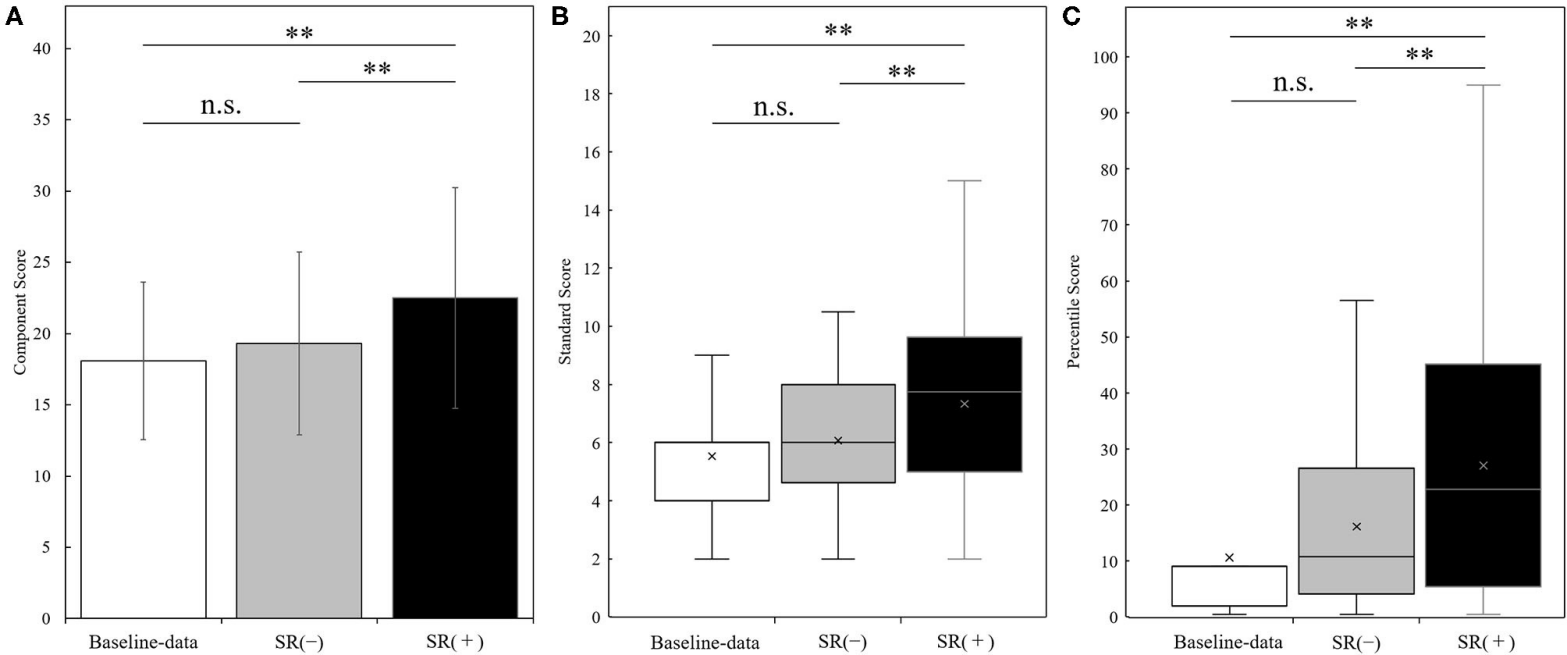
Results of the manual dexterity test (panel **A**: component score; panel **B**: standard score; panel **C**: percentile score) measured at the baseline-data, in the SR-off conditions (SR(-)), and the SR-on conditions (SR(+)) (*N* = 30). Results of the manual dexterity test (Panel **A**: component score, mean ± standard deviation; panel **B**: standard score, boxplot; panel **C**: percentile score, boxplot) in the baseline-data (white bars), the SR-off conditions (SR(-), gray bars), and the SR-on conditions (SR(+), black bars). **p < 0.001; n.s. = not significant.

Figure 3 shows the results of manual dexterity test (component score, standard score, and percentile score) in the baseline-data, SR-on condition (averaged over two blocks), and SR-off condition (averaged over two blocks) in the whole sample (*N* = 30). There was a significant change in component score in the three conditions as shown by repeated measures one-way ANOVA between three conditions [F_(2, 58)_ = 25.385, *p* < 0.001]. *Post-hoc* analysis showed that the component score in the SR-on condition was significantly higher than in the baseline-data and SR-off conditions (SR-on vs. baseline-data, t_(29)_ = −6.196, *p* < 0.001, effect size (r) = 0.75; SR-on vs. SR-off, t_(29)_ = −5.689, *p* < 0.001, effect size (r) = 0.73; all after Bonferroni correction). No statistically significant difference in component score between baseline-data and SR-off condition was observed [t_(29)_ = −1.892, *p* = 0.205, after Bonferroni correction, effect size (r) = 0.33] (Figure 3A).

There was a significant change in standard score in the three conditions as shown by the Friedman test (*p* < 0.001). *Post-hoc* analysis showed that the standard score in the SR-on condition was significantly higher than the standard score in the baseline-data and SR-off condition (SR-on vs. baseline-data, *z* = −4.116, *p* < 0.001, effect size (r) = −0.75; SR-on vs. SR-off, *z* = −3.693, *p* < 0.001, effect size (r) = −0.67; all after Bonferroni correction). No significant difference in standard score between baseline-data and SR-off condition was observed (*z* = −1.873, *p* = 0.183, after Bonferroni correction, effect size (r) = −0.34) (Figure 3B).

There was a significant change in percentile score in the three conditions as shown by the Friedman test (*p* < 0.001). *Post-hoc* analysis showed that the percentile score in the SR-on condition was significantly higher than the percentile score in the baseline-data and SR-off condition (SR-on vs. baseline-data, *z* = −4.108, *p* < 0.001, effect size (r) = −0.75; SR-on vs. SR-off, *z* = −3.667, *p* < 0.001, effect size (r) = −0.67; all after Bonferroni correction). No significant difference in percentile score between baseline-data and SR-off condition was observed (*z* = −2.146, *p* = 0.0957, after Bonferroni correction, effect size (r) = −0.39) (Figure 3C).

## Discussion

The present study showed that manual dexterity under SR-on conditions was significantly improved compared to the baseline-data and to the SR-off conditions. Analysis of test scores throughout the experiment showed that the observed improvement in manual dexterity was not an effect of learning during the experiment but was specifically generated during the SR-on condition.

Hand tactile sensation is a very important factor for accurate and quick manual dexterity (19–22). Previous studies showed that vibrotactile noise stimulation to the wrist with an intensity of 60% of the sensory threshold as used within this study improved fingertip tactility and manual dexterity in the affected limbs of patients with stroke (7, 8, 10). Therefore, the improvement in manual dexterity observed under SR-on condition in the current study may have been due to an improvement in hand tactile sensitivity that is an important component of manual dexterity.

In general, children with DCD have lower ability to effectively use tactile information for movement and have to rely more on visual information (14, 23–25). However, an earlier case study showed that the problem of visual dependence in a child with DCD may be improved as a result of SR stimulation similar to the one used in this study (14). Therefore, the significant improvement in manual dexterity observed during the SR-on condition in the current study may be due both to an increase in tactile sensitivity and a reduction in visual dependence.

In addition, sensory-motor integration is a very important function for manual dexterity in children (26, 27). A previous study showed that the application of SR to healthy young individuals significantly improved sensory-motor integration (17). In addition, one case study showed that SR intervention may improve sensory-motor integration in a child with DCD (14). Therefore, the significant improvement in manual dexterity observed in the SR-on condition may be related to possible improvement in sensory-motor integration.

The advantage of the SR intervention here presented is that children only need to wear a compact SR device and no special efforts are needed to use the device. In the current study, the SR-on and -off conditions were altered and the effects observed during the SR-on conditions were not observed under subsequent SR-off conditions, suggesting that there is no carry-over (retention) effect when switching from SR-on to SR-off. Therefore, future studies will need to investigate the possible influence of the duration of the SR stimulation on carry-over (retention) effects and understand if and under which circumstances the beneficial effects of subthreshold stimulation can be sustained after the end of stimulation. In other words, how long do children with DCD need to wear the device to see a carryover effect? What activities should they perform while they are wearing the device in order to have a carryover effect? These questions need to be clarified. In addition, vibrotactile noise stimulation is non-invasive and unconscious, and the children did not complain of any discomfort (e.g., pain) in this study. However, it is not known whether this stimulation will have a negative effect on the body when it is applied for a long period of time. Future studies are needed to investigate these possible adverse effects. These future studies should help to clarify when clinicians should consider stopping SR administration and the criteria for identifying the inefficacy of SR.

An important limitation of the study is that the children were assigned to two groups according to their order of enrollment, and although there were no significant differences in the baseline data of both groups, they were not completely equal with respect to age and sex. Therefore, in the future, randomized controlled trials should be conducted with larger sample sizes, completely matched for age and sex, and assigned to intervention and no-intervention groups. In addition, the outcome of this study used the M-ABC2, the international standard assessment battery for DCD, but future studies should also use other tools, such as the NEPSY second edition, to assess sensorimotor and visuospatial functions to verify whether manual dexterity improves in the long term.

## Data Availability Statement

The raw data supporting the conclusions of this article will be made available by the authors, without undue reservation.

## Ethics Statement

The studies involving human participants were reviewed and approved by The ethics committee of the Graduate School and Faculty of Health Sciences at Kio University (approval number: R1-22). Written informed consent to participate in this study was provided by the participants' legal guardian/next of kin.

## Author Contributions

SN designed the study, collected and analyzed the data, and wrote the manuscript. MO provided experimental equipment and assisted in collecting data. AN provided evaluation battery and helped with data analyses. AM, EF, TM, SS, and SM supervised the study. All authors read and approved the manuscript.

## Conflict of Interest

The authors declare that the research was conducted in the absence of any commercial or financial relationships that could be construed as a potential conflict of interest.
